# Investigation of nosocomial SARS-CoV-2 transmission from two patients to healthcare workers identifies close contact but not airborne transmission events

**DOI:** 10.1017/ice.2020.321

**Published:** 2021-07-03

**Authors:** Derek J. Bays, Minh-Vu H. Nguyen, Stuart H. Cohen, Sarah Waldman, Carla S. Martin, George R. Thompson, Christian Sandrock, Joel Tourtellotte, Janelle Vu Pugashetti, Chinh Phan, Hien H. Nguyen, Gregory Y. Warner, Bennett H. Penn

**Affiliations:** 1 Division of Infectious Diseases, Department of Internal Medicine, University of California Davis Medical Center, Sacramento, California; 2 Patient Care Services, University of California Davis Medical Center, Sacramento, California; 3 Department of Medical Microbiology and Immunology, University of California, Davis, Davis, California; 4 Division of Pulmonary, Critical Care, and Sleep Medicine, Department of Internal Medicine, University of California Davis Medical Center, Sacramento, California; 5 Division of Infectious Diseases, Department of Internal Medicine, Veterans’ Affairs Northern California Health Care System, Sacramento, California; 6 Division of Infectious Diseases, Department of Internal Medicine, NorthBay Healthcare, Fairfield, California

## Abstract

**Objective:**

To describe the pattern of transmission of severe acute respiratory coronavirus virus 2 (SARS-CoV-2) during 2 nosocomial outbreaks of coronavirus disease 2019 (COVID-19) with regard to the possibility of airborne transmission.

**Design:**

Contact investigations with active case finding were used to assess the pattern of spread from 2 COVID-19 index patients.

**Setting:**

A community hospital and university medical center in the United States, in February and March, 2020, early in the COVID-19 pandemic.

**Patients:**

Two index patients and 421 exposed healthcare workers.

**Methods:**

Exposed healthcare workers (HCWs) were identified by analyzing the electronic medical record (EMR) and conducting active case finding in combination with structured interviews. Healthcare coworkers (HCWs) were tested for COVID-19 by obtaining oropharyngeal/nasopharyngeal specimens, and RT-PCR testing was used to detect SARS-CoV-2.

**Results:**

Two separate index patients were admitted in February and March 2020, without initial suspicion for COVID-19 and without contact or droplet precautions in place; both patients underwent several aerosol-generating procedures in this context. In total, 421 HCWs were exposed in total, and the results of the case contact investigations identified 8 secondary infections in HCWs. In all 8 cases, the HCWs had close contact with the index patients without sufficient personal protective equipment. Importantly, despite multiple aerosol-generating procedures, there was no evidence of airborne transmission.

**Conclusion:**

These observations suggest that, at least in a healthcare setting, most SARS-CoV-2 transmission is likely to take place during close contact with infected patients through respiratory droplets, rather than by long-distance airborne transmission.

Multiple routes of transmission have been postulated for severe acute respiratory coronavirus virus 2 (SARS-CoV-2), including respiratory droplets, airborne particles, and fomites.^1–4^ In particular, the risk of acquiring coronavirus disease 2019 (COVID-19) through inhalation of airborne particles, capable of transmitting infection over long distances, is uncertain, and remains a matter of vigorous debate.^2,5,6^ Given the significant risks of transmission to healthcare workers (HCWs),^7^ defining the degree to which airborne transmission occurs is important for guiding hospital infection control procedures and informing public health policy.

The predominant mode of transmission for most respiratory viruses occurs via large respiratory droplets inoculating mucous membranes.^8^ Respiratory droplets >5 µm in size travel <2 m, remain suspended <20 minutes, and are effectively blocked by surgical masks.^8^ In contrast, smaller droplets evaporate rapidly, and the remaining desiccated droplet nucleus can remain airborne for hours, travel long distances, and require N95 respirators for protection.^8^

For SARS-CoV-2, several laboratory and environmental studies have suggested the possibility of airborne transmission.^5,6,9^ Artificially generated SARS-CoV-2 aerosols were found to be stable, with a half-life of 1.5 h, and viral RNA has been detected on surfaces throughout the rooms of COVID-19 patients, including the ventilation system. The uncertain routes of transmission have led to inconsistent recommendations for infection prevention and appropriate personal protective equipment (PPE) for HCWs. For routine patient care, the WHO recommends contact and droplet precautions,^4^ and only recommends airborne precautions with a respirator in the setting of aerosol-generating procedures (AGPs).^2^ In contrast, the US Centers for Disease Control and Prevention (CDC) expresses a preference for respirators as routine PPE, with droplet and contact precautions being considered an acceptable alternative in the context of supply shortages.^3^

To assess the routes of transmission of SARS-CoV-2, it will be necessary to document the pattern of spread from well-defined exposures. Here, we describe the pattern of nosocomial SARS-CoV-2 transmission from 2 separate patients who were not initially suspected as having COVID-19 and who were cared for without contact, droplet, or airborne precautions.

## Methods

### Contact investigations

Investigation 1A reviews the contact investigation for patient 1 at hospital A, a community hospital. The HCWs wore neither surgical masks nor eye protection, and they were risk stratified based on examination of the medical record and subsequent phone interviews as follows: high risk: nose or mouth exposed during intubation or bronchoscopy; moderate: nose or mouth exposed for >2 minutes; and low: nose or mouth exposed <2 minutes.

Investigation 1B and investigation 2 were completed at hospital B, a university medical center. In both instances, hospital B undertook active case finding, with a combination of electronic medical record (EMR) tracing to identify all HCWs who entered the index patient’s record, as well as surveys conducted by each unit manager to identify any HCWs that may have entered the room without EMR contact. Exposed HCWs filled out structured surveys regarding PPE, and types of contact, including AGPs. In addition, any HCWs with an influenza-like illness (ILI) underwent testing as per hospital policy, including HCWs not directly involved in the patient’s clinical care. A number of asymptomatic HCWs were also tested because they were deemed higher risk for transmitting to patients, including respiratory therapists and all members of the oncology team.

Exposed HCWs at hospital B were risk stratified with the following designations: patient source controlled with mask or intubation (Con+/−), and PPE with surgical mask (M+/−) and eye protection (E+/−). Risk level was defined as: high: (Con−M−E−), moderate (Con−M−E+ or Con−M+E−), and low (Con−M+E+ or Con+M+E−). No asymptomatic patients were tested, and no patients developed an ILI that triggered SARS-CoV-2 testing. Using the criteria of hospital B, all exposures at hospital A would have been considered high risk because no HCWs wore masks, eye protection, or gowns. For case 1, testing relied on oropharyngeal or nasopharyngeal swabs, with RT-PCR performed at the California Department of Public Health (CADPH). For case 2, only nasopharyngeal swabs were used, and specimens were tested on-site by hospital B using a validated assay on an ABI StepOnePlus instrument. The institutional review board (IRB) at hospital B deemed that IRB approval and informed consent were unnecessary due to the quality improvement origins of the work.

### Statistical analysis

The Fisher exact test was used to assess the association between specific high-risk procedures and a positive SARS-CoV-2 using RT-PCR.

## Case 1

### Clinical course at hospital A

A previously healthy woman aged in her 40s, who would later be deemed the first case of community-acquired COVID-19 diagnosed in the United States, presented to a local hospital with 48 hours of subjective fever, dry cough, nausea, and vomiting.^10^ Upon presentation she was febrile, tachycardic, and hypotensive, and her chest x-ray showed a focal consolidation. The patient was admitted to the general medical-surgical ward with a diagnosis of community-acquired pneumonia, and intravenous antibiotics treatment began. Over 2 days, she became increasingly hypoxic, requiring oxygen through high-flow nasal canula, and her chest x-ray showed progressive disease. By day 3, she required noninvasive mechanical ventilation, and she eventually transferred to the intensive care unit (ICU) and endotracheally intubated. The patient underwent an initial abbreviated bronchoscopy followed by a second longer diagnostic bronchoscopy that showed only bloody secretions. The patient’s hypoxemia worsened, and she was subsequently transferred on day 4 to hospital B for consideration of extracorporeal membrane oxygenation (ECMO).

### Clinical course after transfer to hospital B

Extensive additional evaluation for the etiology of acute respiratory distress syndrome (ARDS) was completed and unrevealing. Despite broad-spectrum antimicrobials, she had persistent fever and hypoxemic respiratory failure, although she did not require ECMO. At the time of her illness, SARS-CoV-2 testing was only available through the CDC. Although she had no travel history that qualified her for SARS-CoV-2 testing, her severe presentation and unrevealing diagnostic evaluation suggested the possibility of COVID-19. On day 5 of her course at hospital B, a nasopharyngeal swab for SARS-CoV-2 was sent to the CDC, which returned positive. Remdesivir was obtained through a compassionate use authorization, the patient slowly improved, and ultimately, was discharged home after ~1 month.

### Case 1 contact investigation—hospital A

Because COVID-19 was not initially suspected, a large number of HCWs was exposed to the index case at hospital A without PPE; many of the details of this contact investigation have been recently reported in a CDC-issued *Morbidity and Mortality Weekly Report*.^11^ No contact, droplet, or airborne precautions were used. The patient initially spent time on the combined medical-surgical ward with 29 beds and staffed by 8 RNs, several physicians, and ancillary staff. She was then was transferred to the ICU, which has 6 rooms. The patient’s room for intubation and bronchoscopy measures 22.76 m^2^ (245 feet^2^), was not negative pressure, and has net air exchange of 2.83 m^3^/min (100 feet^3^/min).

**Fig. 1.**
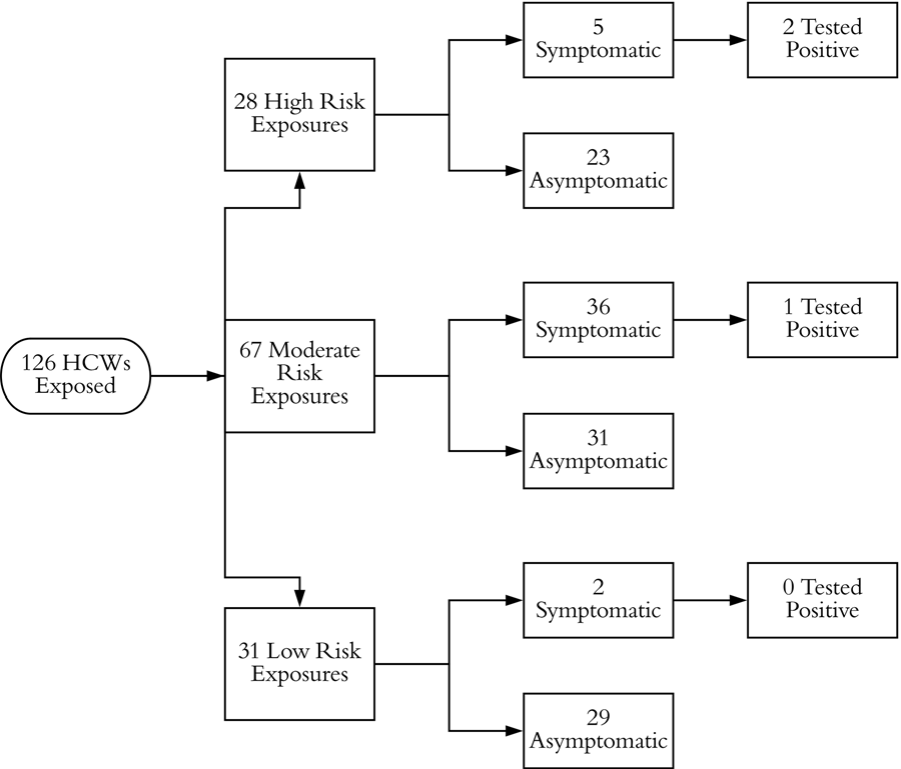
Stratification of exposed healthcare workers (HCWs) for case 1 at hospital A.

**Fig. 2.**
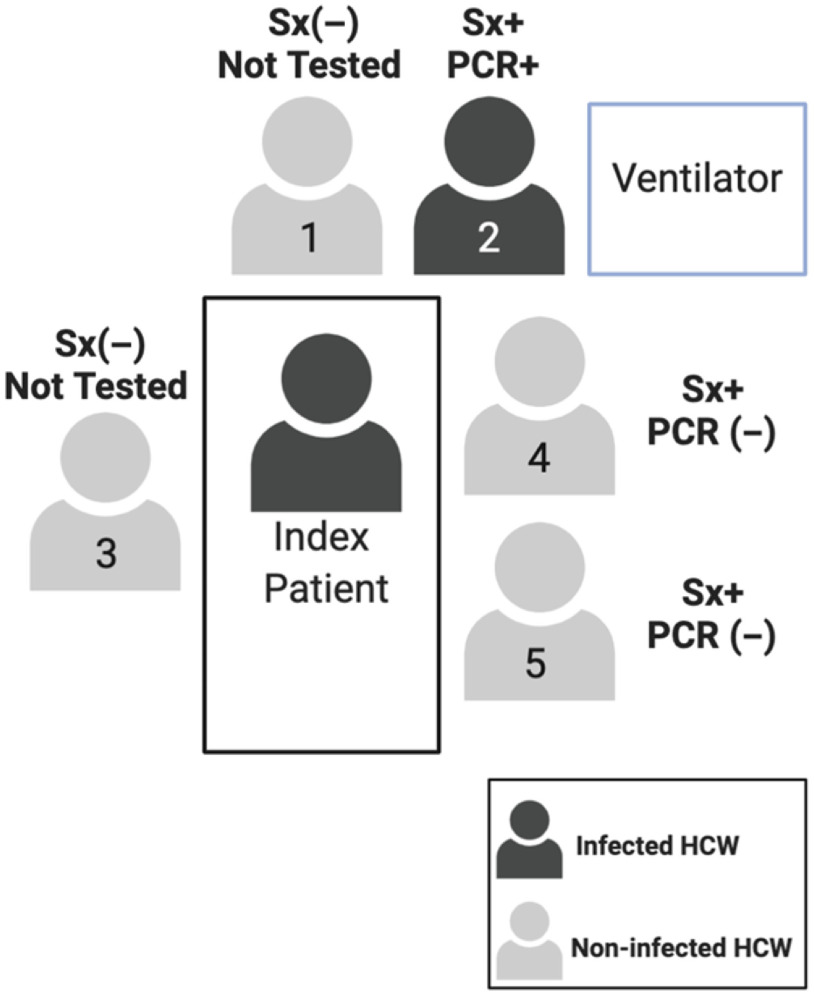
Description of staff at endotracheal intubation for case 1 at hospital A. Note. Sx+, symptomatic; Sx (−) asymptomatic; PCR +/(−) denotes SARS-CoV-2 test result.

The contact investigation at hospital A determined that, in total, 126 HCWs were exposed, of whom 28 HCWs were deemed high risk, 67 were deemed moderate risk, and 31 were deemed low risk. Of these 126 HCWs, 43 developed an ILI. These 43 HCWs were tested, and 3 HCWs tested positive (Fig. 1). All 3 had provided direct patient care with close contact for several days, and were present for AGPs without masks or eye protection. Two SARS-CoV-2–positive HCWs were direct providers on the ward and were present while the patient received oxygen by high flow nasal cannula or noninvasive positive-pressure ventilation, the third HCW provided care both on the medical ward and the ICU and was present for the intubation and bronchoscopies (Fig. 2). In summary, although 43 HCWs underwent testing, the infected HCWs all had had prolonged direct contact with the patient, including during AGPs.

### Case 1 contact investigation—hospital B

**Fig. 3.**
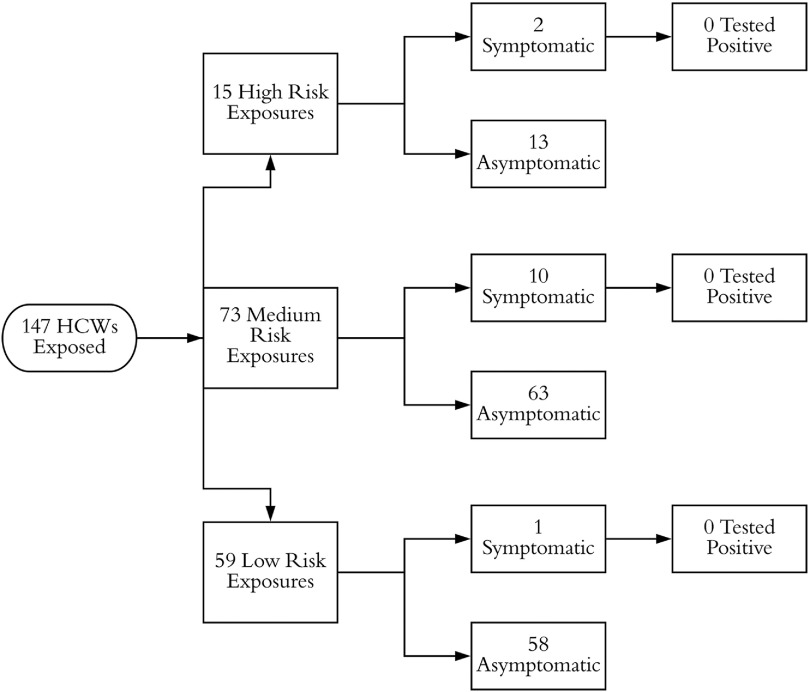
Stratification of exposed healthcare workers (HCWs) for case 1 at hospital B.

Upon transfer, droplet and contact precautions were instituted, and respiratory pathogen PCR testing was performed. On hospital day 3, when the respiratory pathogen panel returned negative, droplet and contact precautions were discontinued. On hospital day 5, when it became possible to test for SARS-CoV-2, airborne precautions were instituted. Testing returned positive, and airborne precautions were continued until hospital day 23, when she had 2 negative SARS-CoV-2 nasopharyngeal swabs from consecutive days return negative, and airborne, droplet, and contact precautions were discontinued. Prior to the institution of airborne isolation, 147 HCWs at hospital B were exposed to the patient (Fig. 3). There were 15 high-risk exposures, 73 medium-risk exposures, and 59 low-risk exposures. All of the high- and medium-risk HCWs (88 HCWs) were isolated from work for 14 days. Ultimately, 13 employees developed ILI symptoms and were tested for SARS CoV-2 using RT-PCR, but all tested negative.

## Case 2

### Clinical course

A previously healthy man aged in his 60s presented to a local hospital with dyspnea. He was found to have a deep vein thrombosis with pulmonary emboli and was noted to have a leukocyte cell count of 69,000 cells/mm^3^ with myeloblasts. He was transferred to hospital B, and on day 2, he developed progressive hypoxemic respiratory failure; he was intubated on day 3. Bone marrow biopsy confirmed AML, and his course was complicated by the presumed sequalae of leukostasis with disseminated intravascular coagulation, acute left middle cerebral artery infarct, subarachnoid hemorrhage, acute kidney injury, and splenic rupture. He remained persistently febrile and underwent an unrevealing diagnostic bronchoscopy, and on day 14 the infectious diseases service was consulted. A nasopharyngeal swab for SARS-CoV-2 testing was obtained, and droplet and contact precautions, with airborne precautions for AGPs were instituted. SARS-CoV-2 testing returned positive on day 15, with a cycle-threshold (Ct) value of 25. The patient then developed central venous catheter–associated bloodstream infection with septic shock and despite intravenous antibiotics and catheter removal, he continued to decline. The family ultimately opted to pursue comfort-focused care, and the patient died on hospital day 30.

### Case 2 contact investigation

The patient was originally admitted to the oncology unit, which consists of 25 neutral-pressure rooms with 35 beds and is staffed by 13 nurses per shift and 5–6 physicians on the oncology team. He was transferred to the medical ICU, which consists of 16 single-room beds, each with an assigned nurse, as well as 2 teams with 6–7 physicians each and a variable number of respiratory therapists. The room in which the patient was intubated measures 15.33 m^2^ (165 feet^2^) and has 15 air exchanges per hour but is not negative pressure relative to the unit. Between hospital day 3 and day 15, there was some degree of source control; his ventilator was fitted with closed-circuit suctioning an in-line high-efficiency particulate air (HEPA) filter.

**Fig. 4.**
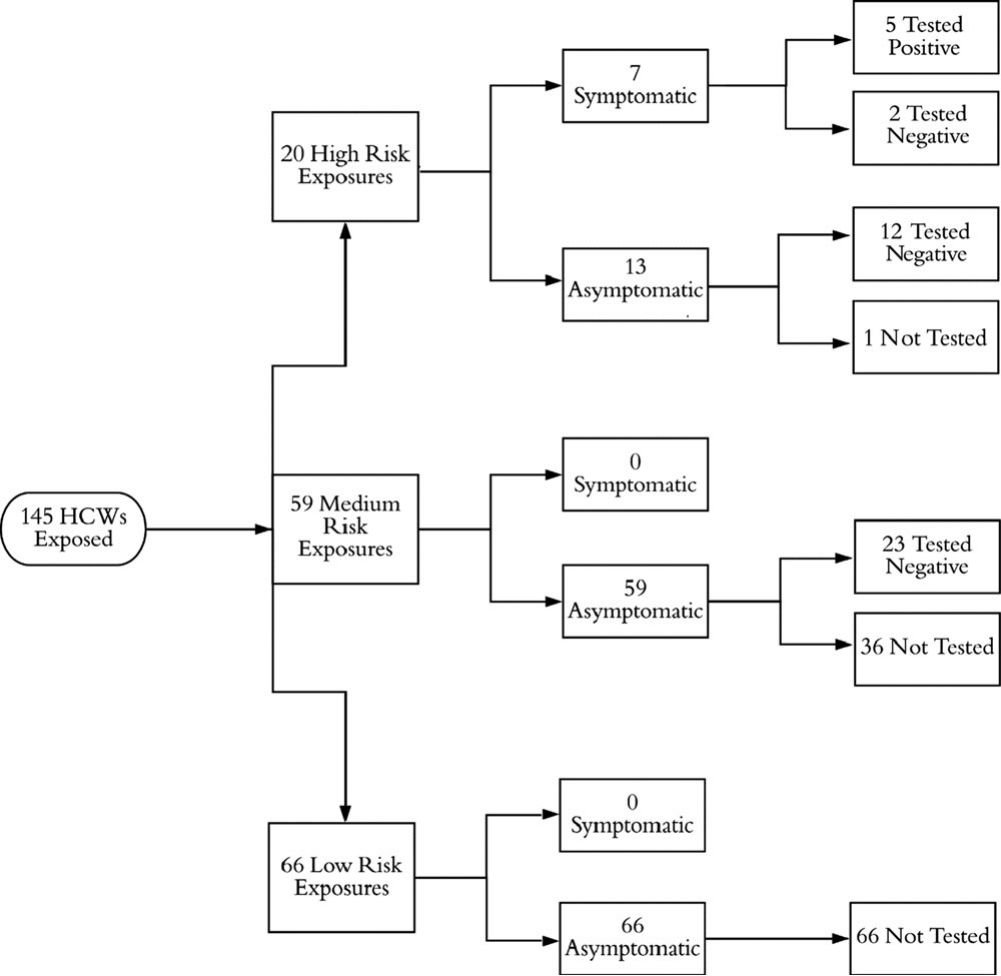
Stratification of exposed healthcare workers (HCWs) for case 2.

**Fig. 5.**
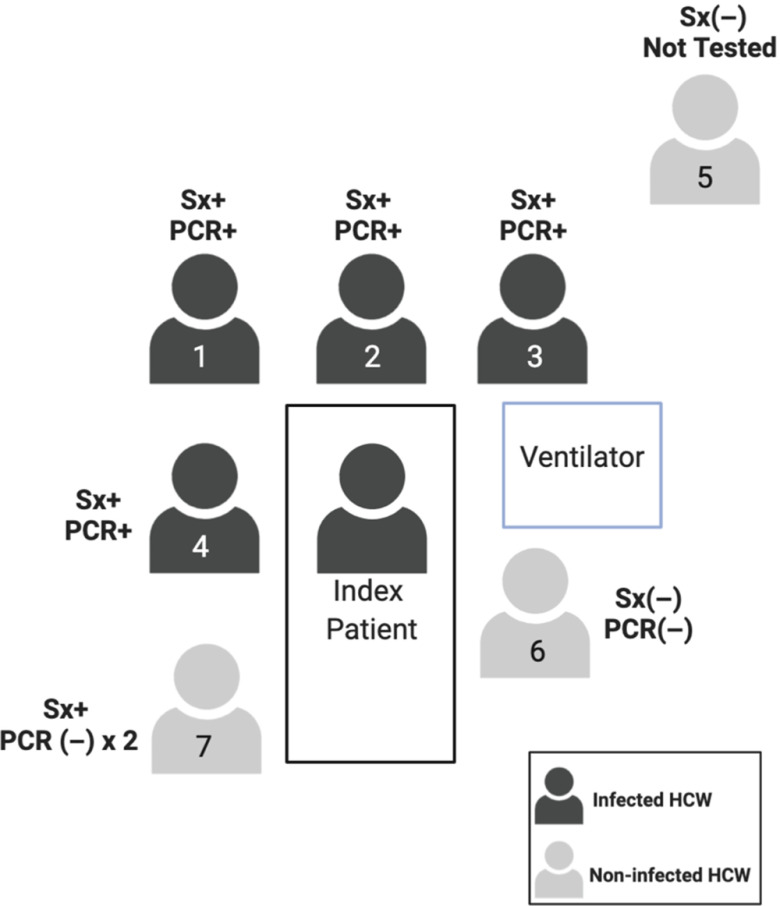
Description of staff present during intubation of case 2. Note. Sx+, symptomatic; Sx(−) asymptomatic; PCR +/(−) denotes SARS-CoV-2 test result.

Overall, 145 HCWs were identified as having exposure to the index patient, with 5 confirmed infections and 2 possible infections (Fig. 4). Of the 145 HCWs, 7 developed ILI symptoms and all were at the bedside for AGPs without adequate PPE. The patient underwent 2 significant AGPs: endotracheal intubation on day 3 and bronchoscopy on day 11, with neither airborne nor droplet precautions in place. Most transmission events were associated with the endotracheal intubation; 4 of the 7 HCWs present for the procedure tested positive for SARS-CoV-2 (*P* < .001) (Fig. 5). The individual performing the procedure wore a surgical mask without eye protection, and the remaining HCWs wore neither masks nor eye protection (Table 1). A fifth HCW, who was at neither the bronchoscopy nor intubation, also developed symptoms, but this HCW had direct patient contact for several days without PPE and assisted in transferring the patient between ventilators, which necessitated a break in the closed ventilation circuit. All HCWs who tested positive developed symptoms within a 72-hour window. Two additional HCWs who had direct patient contact without PPE during AGPs, whom we consider possible cases, developed high fevers and cough but tested negative for SARS-CoV-2 twice each. Interestingly, we identified no transmission during the bronchoscopy when all HCWs wore surgical masks and eye protection. Overall 7 HCWs were present, and the 2 providers who performed the bronchoscopy wore surgical masks with eye protection, a gown, and gloves. Everyone else wore a surgical mask with eye protection (Table 1). Thus, in summary, although a number of HCWs became infected by the index case, they all had direct contact with the patient and were present during AGPs without sufficient PPE.

**Table 1.** Summary of PPE and SARS-CoV-2 Testing for Case 2 Procedures

**Intubation**		
Staff 1	Gloves, no mask or eye protection	Symptomatic, PCR +
Staff 2^a^	Gloves and mask without eye protection	Symptomatic, PCR +
Staff 3	Gloves, no mask or eye protection	Symptomatic, PCR +
Staff 4	Gloves, no mask or eye protection	Symptomatic, PCR +
Staff 5	Gloves, no mask or eye protection	Symptomatic, PCR (−) × 2
Staff 6	Gloves, no mask or eye protection	Asymptomatic, not tested
Staff 7	Gloves, no mask or eye protection	Asymptomatic, not tested
**Bronchoscopy**		
Staff 7^a^	Mask, eye protection, gown, gloves	Asymptomatic, not tested
Staff 8^a^	Mask, eye protection, gown, gloves	Asymptomatic, not tested
Staff 9	Mask, eye protection	Symptomatic, PCR (−)
Staff 10	Mask, eye protection	Asymptomatic, not tested
Staff 11	Mask, eye protection	Asymptomatic, not tested
Staff 12	Mask, eye protection	Asymptomatic, not tested

Note. Mask indicates surgical mask; PCR +/(−) denotes SARS-CoV-2 test result.

a Denotes staff performing the procedure.

## Discussion

The cases described here, and the pattern of spread to exposed HCWs, provide important insight into the transmissibility of SARS-CoV-2 in a healthcare setting. Both patients were in the hospital for several days before COVID-19 was suspected, without contact, droplet, or airborne precautions in place, and both patients underwent multiple AGPs without negative-pressure isolation rooms. The hypothesis that SARS-CoV-2 is airborne transmissible would predict widespread infection of HCWs or other patients during this time, unconstrained by the 2-m radius that large respiratory droplets travel. Indeed, this is precisely the pattern seen with well-established airborne-transmitted agents such as tuberculosis and measles, in which patients cared for without negative-pressure isolation have triggered multiple outbreaks, with infection spreading to HCWs and other patients throughout a unit who had no direct contact with the index case.^13–15^

For the cases described here, this did not occur. Although 8 HCWs were infected, transmission occurred exclusively among HCWs that were at the patient’s bedside without contact and droplet PPE. No apparent transmission to HCWs or patients occurred elsewhere on the units, including an oncology ward housing a large number of immunocompromised patients. These findings are much more consistent with transmission by respiratory droplets than by airborne transmission.^16,17^ This idea is further supported by 3 other recent reports, each of individual patients with unsuspected COVID-19 in which an absence of airborne transmission was similarly documented.^18–20^

Several possibilities exist to reconcile the theoretical concern for airborne transmission raised by other studies^5,6,21^ with our contact investigations showing no apparent airborne transmission. Although artificially generated SARS-CoV-2 airborne particles are quite stable and SARS-CoV-2 RNA can be detected throughout COVID-19 patient rooms, no infectious virus could be recovered from the rooms, suggesting that the viral RNA might be from replication intermediates or noninfectious virions. However, it is impossible to exclude the possibility that virions isolated from surfaces were initially infectious but had degraded prior to sample collection. An alternative explanation may lie in the dose of SARS-CoV-2 necessary to establish infection. The minimum infectious dose varies dramatically between respiratory pathogens, with <10 bacilli needed to establish *M. tuberculosis* infection, but >500 virions needed for echovirus.^20^ The minimum infectious dose for SARS-CoV-2 in humans is unknown, but a likely explanation for a failure of SARS-CoV-2 airborne particles to efficiently transmit infection over long distances may simply be that the number of inhaled virions is insufficient to establish infection.

Our observations did not discriminate between close-range transmission by large respiratory droplets that can be effectively blocked by surgical masks and eye protection versus close-range transmission by small droplets and droplet nuclei that penetrate surgical masks. The potential importance of these small particles was highlighted during the 2003 SARS outbreak, when high-risk AGPs such as intubation and cardiopulmonary resuscitation were analyzed. During these procedures, HCWs with close patient contact became infected, even when droplet precautions were in place, with endotracheal intubation having an odds ratio of 13.^21^ Because the HCWs who became infected in our study wore neither droplet nor airborne protective equipment, we cannot assess the relative degree of protection that would have been provided by droplet precautions relative to N95 respirators.

Our report has several limitations. Most importantly, a large number of HCWs was exposed, with 421 individuals identified by contact tracing, we were limited by having only 2 index cases, and we had viral load information for only 1 case. This patient had a Ct value of 25, approximately the median value of other studies.^24^ Possibly, patients with higher viral loads more readily transmit infection via airborne particles. In addition, at the time these hospital outbreaks occurred, in February and March 2020, testing infrastructure was still very limited, and systematic testing of all asymptomatic HCWs or patients on each unit was not possible. Given the likelihood of asymptomatic COVID-19 cases,^25^ we cannot exclude occult transmission leading to asymptomatic secondary cases. However, no additional cases were detected amongst the 35 asymptomatic HCWs who were able to be tested, and all HCWs who developed ILI symptoms were tested for SARS-CoV-2, regardless of whether they had had contact with an index patient. Finally, the sensitivity of a single nasopharyngeal test has been reported at 63%,^22^ so some HCWs with COVID-19 may have gone undetected. However, limitations in test sensitivity would apply to HCWs both with and without direct contact and would not be expected to bias the overall distribution of cases. In summary, our findings suggest that, at least in a healthcare setting, most SARS-CoV-2 transmission likely takes place during close contact with infected patients rather than by long-distance airborne transmission.
